# Heat-shock protein 70-dependent dendritic cell activation by 5-aminolevulinic acid-mediated photodynamic treatment of human glioblastoma spheroids *in vitro*

**DOI:** 10.1038/bjc.2011.327

**Published:** 2011-08-23

**Authors:** N Etminan, C Peters, D Lakbir, E Bünemann, V Börger, M C Sabel, D Hänggi, H-J Steiger, W Stummer, R V Sorg

**Affiliations:** 1 Department of Neurosurgery, Heinrich-Heine-University Hospital, Bldg. 13.71, Moorenstrasse 5, Düsseldorf 40225, Germany; 2 Institute for Transplantation Diagnostics and Cell Therapeutics, Heinrich-Heine-University Hospital, Moorenstrasse 5, Düsseldorf 40225, Germany; 3 Department of Dermatology, Heinrich-Heine-University Hospital, Moorenstrasse 5, Düsseldorf 40225, Germany; 4 Department of Neurosurgery, University of Münster, Albert-Schweitzer-Strasse 33, Münster 48149, Germany

**Keywords:** aminolevulinic acid, glioma, photodynamic therapy, dendritic cells, HSP-70

## Abstract

**Background:**

T-cell responses contribute to the anti-tumoural effect of photodynamic therapy (PDT). For such responses to occur, dendritic cells (DCs) have to migrate to the tumour, take up tumour antigens and respond to danger signals with maturation, before they engage in T-cell activation. Here, we have studied the effect of 5-aminolevulinic acid (ALA)-mediated PDT on DCs *in vitro* in a human spheroid model of glioblastoma (GB).

**Methods:**

Spheroids of the GB cell lines U87 and U251 were treated with ALA/PDT, and effects on attraction, uptake of tumour antigens and maturation of DCs were studied. To block heat-shock protein-70 (HSP-70) on the spheroids, neutralising antibodies were used.

**Results:**

5-Aminolevulinic acid /PDT-treated GB spheroids attracted DCs that acquired tumour antigens from the spheroids effectively. Moreover, co-culture with ALA/PDT-treated spheroids induced DC maturation as indicated by the upregulation of CD83 and co-stimulatory molecules as well as increased T-cell stimulatory activity of the DCs. Heat-shock protein-70 was upregulated on the spheroids after ALA/PDT treatment. Uptake of tumour antigens and DC maturation induced by the ALA/PDT-treated spheroids were inhibited when HSP-70 was blocked.

**Conclusion:**

ALA/PDT treatment of glioma spheroids promotes the three initial steps of the afferent phase of adaptive immunity, which is at least partially mediated by HSP-70.

Photodynamic therapy (PDT) is a promising approach in the treatment of various tumours (Dolmans *et al*, 2003). It is based on the preferential accumulation of a photosensitiser in tumour cells and its excitation with light of a defined wavelength. This excitation initiates a photochemical reaction, resulting in the generation of reactive oxygen species within the tumour cells, for example, singlet oxygen, which cause secondary reactions leading to cell death. Photodynamic therapy results either in apoptosis or necrosis of the tumour cells, depending on the photosensitiser and its intracellular localisation, the cell type, oxygen concentration and PDT dose (Castano *et al*, 2006).

Although PDT-induced phototoxicity kills tumour cells directly, it is well established that there is also an immunological component to the anti-tumoural effect of PDT (Dolmans *et al*, 2003; Castano *et al*, 2006). Reduced efficacy of PDT in immunodeficient mice lacking T cells (Korbelik *et al*, 1996; Hendrzak-Henion *et al*, 1999; Preise *et al*, 2009) indicates a pivotal role of T cells. This is further supported by adoptive transfer experiments (Korbelik and Dougherty, 1999; Preise *et al*, 2009) and lymphocyte-depletion studies (Hendrzak-Henion *et al*, 1999; Korbelik and Cecic, 1999; Korbelik and Dougherty, 1999), identifying CD8^+^ cytotoxic T cells as main effectors. However, for such T-cell responses to occur, tumour antigens have first to be taken up by immature dendritic cells (DCs), danger signals have to induce DC maturation, associated with migration of DCs to local lymph nodes and mature DCs in the lymph nodes have to present the tumour antigens to the specific T cells in the context of adhesion, co-stimulatory and other accessory molecules, either directly or after antigen transfer to other DCs (Ueno *et al*, 2010).

Photodynamic therapy generates an altered tumour microenvironment that appears to promote this afferent phase of adaptive immunity. Tumour cells undergoing necrosis or apoptosis release tumour antigens that can be taken up by DCs (Green *et al*, 2009). Moreover, immediately after PDT an inflammatory response develops providing danger signals such as pro-inflammatory cytokines. Together with stress proteins like heat-shock proteins (HSP) induced on the surface or released by the tumour cells after PDT and the dead tumour cells themselves, these cytokines may promote DC maturation (Gollnick *et al*, 1997; Dolmans *et al*, 2003; Jalili *et al*, 2004; Korbelik *et al*, 2005; Castano *et al*, 2006). In several mouse models for extracranial tumours, attraction of DCs towards PDT-generated vaccines (Korbelik and Sun, 2006), uptake of tumour antigens by DCs (Jalili *et al*, 2004) and PDT-induced DC maturation (Gollnick *et al*, 2002; Jalili *et al*, 2004) have been reported. No such studies have been performed on human cells or cranial tumours.

Glioblastoma (GB) is the most frequent and aggressive malignant primary brain tumour (Fisher *et al*, 2007). Despite a multimodal therapeutic approach combining maximal cytoreductive surgery with fractionated radiotherapy and alkylating temozolomide chemotherapy, prognosis remains dismal: median survival of patients is 14.6 months, with a 2-year survival rate of 27.2% (Stupp *et al*, 2009). After 3 years, most patients have relapsed and median survival after relapse is 6.2 months (Stupp *et al*, 2009). As a consequence, PDT in combination with highly selective photosensitisers, for example, 5-aminolevulinic acid (ALA), is gaining clinical interest for the treatment of patients with GB (Beck *et al*, 2007; Eljamel *et al*, 2008; Stummer *et al*, 2008).

5-Aminolevulinic acid is an intermediate of the haem biosynthesis pathway. In many tumour cells including GB, an excess of the exogenous pro-drug ALA results in the accumulation of protoporphyrin IX, probably mainly due to low ferrochelatase activity (Krammer and Plaetzer, 2008; Teng *et al*, 2011). If exposed to a wave length of 400 nm, this preferential accumulation of protoporphyrin IX in tumour cells allows their intraoperative identification during fluorescence-guided surgery. In GB, the extent of resection can thereby be increased significantly, leading to improved progression-free survival of patients (Stummer *et al*, 2006). When exposed to 635 nm, protoporphyrin IX acts as a potent photosensitiser, which can be exploited for intraoperative as well as stereotactic PDT of GB (Beck *et al*, 2007; Eljamel *et al*, 2008; Stummer *et al*, 2008). In a patient with non-resectable, recurrent GB, who had failed multimodal therapy, stereotactic ALA/PDT resulted in a long-sustaining response (Stummer *et al*, 2008). Moreover, in a recent phase III study combining ALA- and photofrin-mediated fluorescence-guided resection and PDT, a benefit in survival and time to progression was observed (Eljamel *et al*, 2008). The phototoxic damage caused by ALA/PDT, however, can extend only up to a depth of 4 mm in cerebral tissue (Olzowy *et al*, 2002), and therefore would not reach the glioma cells in the more distant infiltration zone of the tumour. Hence, even for cranial tumours like GB, an immunological effector mechanism may be part of the action of ALA/PDT.

Glioblastoma can be a target of anti-tumoural immune responses (Van Gool *et al*, 2009), and PDT causes inflammatory conditions at extracranial sites like those that are needed to attract DCs to the brain (Zozulya *et al*, 2007). Moreover, despite a lack of typical lymph drainage, DCs from the brain appear to acquire antigens there and migrate to the cervical lymph nodes to initiate CD8^+^ T-cell responses, which is well documented, for example, for neuroinflammatory conditions (Karman *et al*, 2004a, 2004b; Hatterer *et al*, 2008; Steel *et al*, 2009). However, little is known about such mechanisms in GB and about the influence of ALA/PDT treatment of gliomas on the afferent phase of adaptive immunity. Therefore, we have studied in a human tumour spheroid model the effects of ALA/PDT on attraction, tumour antigen uptake and maturation of DCs, and provide evidence that ALA/PDT *in vitro* promotes all of these activities.

## Materials and methods

### Generation of GB spheroids

The human GB cell lines U87 and U251 were maintained in DMEM medium (Lonza, Verviers, Belgium) supplemented with 10% foetal calf serum, 100 U ml^−1^ penicillin, 100 *μ*g ml^−1^ streptomycin and 2 mM L-Gln (all from Lonza) at 37 °C and 5% CO_2_ in tissue culture flasks (Greiner, Nürtingen, Germany). To induce glioma spheroid formation, cells were harvested, and 2 × 10^6^ cells were plated in 20 ml supplemented DMEM medium without phenol red in agar-coated T75 tissue culture flasks. After 3 to 4 days of culture, spheroids with a diameter of 200 *μ*m had formed.

### ALA/PDT treatment of GB spheroids

Spheroid cultures were incubated with 12.5 *μ*g ml^−1^ ALA (Merck, Darmstadt, Germany) for 4 h at 37 °C and 5% CO_2_ as described (Etminan *et al*, 2011). A measure of 10 ml of culture was transferred into 10-cm Petri dishes, spheroids collected under microscopic control and transferred (25 spheroids per well) into agar-coated flat-bottom 96-well plates (Greiner) containing 100 *μ*l per well of DMEM medium without phenol red. For laser-light-only controls, spheroids not treated with ALA were transferred into 96-well plates. Exposure of spheroids to laser light was performed for 625 s with 1 W (equivalent to 25 J s^−1^) using a Ceralas 633 nm PDT diode laser (Biolitec, Jena, Germany). For ALA-only controls, spheroids were treated with ALA, but not exposed to laser light. After laser light exposure, spheroids were used directly for the experiments. Under these conditions, viability of cells was 50% after 16 h of tissue culture as determined by WST-1 assay, and TUNEL staining indicated apoptosis in about 60% of cells (Etminan *et al*, 2011).

### Generation of DCs

Buffy coats were obtained from healthy individuals after informed consent. CD14^+^ monocytes were immunoselected from PBMCs using LS separation columns on a VarioMACS (Miltenyi Biotec, Bergisch Gladbach, Germany) as described (Rapp *et al*, 2006). Differentiation and maturation of DCs from monocytes was performed following a protocol adapted from Zhou and co-workers (Zhou and Tedder, 1996; Rapp *et al*, 2006). Briefly, CD14^+^ monocytes were cultured in 24-well plates (Greiner) at 1 × 10^6^ cells per ml and 2 ml per well in serum-free CellGroDC medium (Cellgenix, Freiburg, Germany) supplemented with 1000 U ml^−1^ GM-CSF (Leukine, Berlex, Richmond, CA, USA) and 1000 U ml^−1^ IL-4 (Cellgenix). After 3 days, half of the medium was replaced by fresh medium containing cytokines. Immature CD14^−^DCs were harvested after 6 days of culture. To induce DC maturation, 1000 U ml^−1^ TNF*α* (Cellgenix), GM-CSF and IL-4 were added to immature DCs on day 6, and cultures were continued for 3 days.

The influence of GB spheroids on DC maturation was studied in co-cultures. In all, 25 untreated spheroids or spheroids that had been treated with ALA (ALA-only) or exposure to laser light (laser-light-only) or both (ALA/PDT) were added to the DC cultures on day 6 and cultures continued for 3 days before analysis. To study the effect of blocking HSP-70 on glioma spheroids, the spheroids were pre-incubated with goat-anti-human HSP-70 polyclonal IgG antibody (1 :20  Santa Cruz, Heidelberg, Germany) before the co-cultures were initiated.

### Flow cytometry and monoclonal antibodies

To assess uptake of tumour material, treated and untreated spheroids were labelled with CFSE (Molecular Probes/Invitrogen, Karlsruhe, Germany) and co-cultured with immature DCs. After 16 h of co-culture, DCs were labelled with anti-HLA-DR monoclonal antibody (mAb) and uptake of tumour material by HLA-DR^+^ DCs was determined by flow cytometry on an FACS Canto using the DIVA software (BD Biosciences, Heidelberg, Germany), identifying antigen uptake by the appearance of HLA-DR/CFSE double-positive DCs within living cells identified based on forward- *vs* side-scatter characteristics. At least 10 000 living cells were acquired in each experiment. To study the effect of blocking HSP-70 on antigen uptake, the spheroids were pre-incubated with goat-anti-human HSP-70 polyclonal IgG antibody before assessing antigen uptake by flow cytometry.

The following mAbs were used for immunostainings: PE-conjugated CD14 (RMO52), CD83 (HB15a) and FITC-conjugated CD80 (MAB104) and CD83 (HB15a) specific mAb from Beckman-Coulter (Krefeld, Germany); PE-conjugated HLA-DR (G46-6) and FITC-conjugated CD40 (5C3) and CD86 (FUN-1) specific mAb from BD Biosciences; and PE-conjugated goat polyclonal IgG specific for HSP-70 (K-20) from Santa Cruz.

### Migration assay

To assess migration of immature DCs towards spheroids, a transwell assay was used. Spheroids, CellGroDC medium (negative control) and medium containing 40 ng ml^−1^ CCL3 (positive control; R&D Systems, Wiesbaden, Germany) were transferred into a 24-well plate (500 *μ*l per well). Transwell inserts (8 *μ*m pore size; Greiner) were coated with 100 *μ*l fibronectin (5 *μ*g ml^−1^; Sigma/Aldrich, Seelze, Germany) for 1 h at room temperature, placed in the 24-well plate containing the migration targets and immature DCs in CellGroDC medium (50 000 DCs/200 *μ*l per well) were added into the insert. After 16 h of incubation at 37 °C and 5% CO_2_, cells were fixed with methanol and migrated DCs stained with Fields solution (Alfa Aesar, Karlsruhe, Germany) before microscopically determining the number of migrating cells. Counting of cells was performed automatically using the ImageJ software (http://rsbweb.nih.gov/ij/).

### Allogeneic mixed leukocyte reaction

Graded doses of immature DCs, TNF*α*-matured DCs or immature DCs, which had been co-cultured for 3 days with ALA/PDT-treated or untreated spheroids, were co-cultured with 10^5^ allogeneic T cells, which were obtained by negative selection from buffy coats as described (Sorg *et al*, 2001). After 5 days of co-culture, cells were pulsed with bromodeoxyuridine for 16 h, and bromodeoxyuridine uptake was determined using the bromodeoxyuridine enzyme-linked immunosorbent assay kit as recommended by the manufacturer (Roche Diagnostics, Mannheim, Germany).

### Statistics

If not stated otherwise, all data are presented as mean±s.e.m. Statistical analysis was performed with the Graph Pad Prism Software Version 5.01 (GraphPad, San Diego, CA, USA). Statistical significance was evaluated with the Student's *t*-test or the two-way ANOVA test.

## Results

### ALA/PDT-treated spheroids attract immature DCs

**Figure 1:**
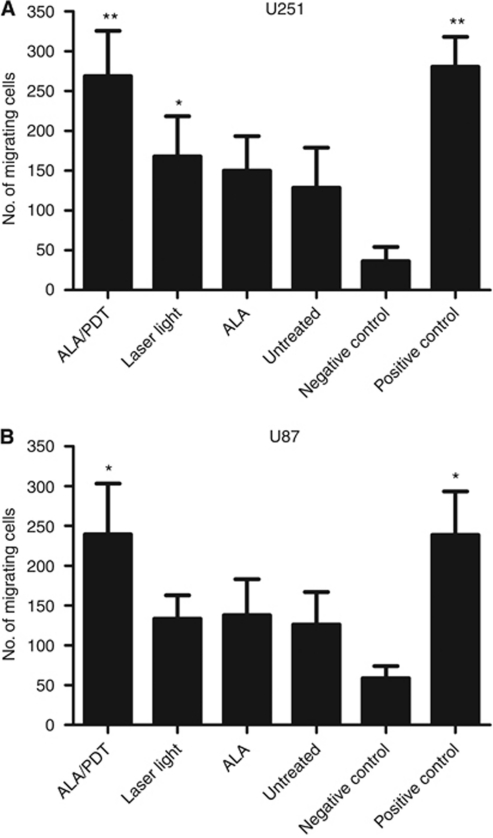
Chemoattractive activity of glioma spheroids for immature DCs. Immature DCs were assessed for migration towards ALA/PDT-treated, ALA-only-treated, laser-light-only exposed and untreated U251 (**A**) and U87 spheroids (**B**). Culture medium served as negative control and medium supplemented with CCL3 as positive control. Data are presented as mean±s.e.m for *n*=4. Statistically significant differences compared to negative control cultures in the absence of spheroids are indicated; ^**^P⩽0.01, ^*^P⩽0.05.

Attraction of immature DCs to ALA/PDT-treated spheroids or control spheroids (untreated, ALA-only- or laser-light-only-treated) was assessed in a transwell migration assay (Figure 1). The immature DCs showed significant migration towards ALA/PDT-treated U251 (269±57 *vs* 36±18 migrating cells; *n*=4, *P*=0.0079) and U87 spheroids (239±64 *vs* 59±15 migrating cells; *n*=4, *P*=0.0337) compared to medium controls. Control spheroids showed no or only weak attraction of immature DCs.

### Enhanced uptake of tumour material by immature DCs after ALA/PDT treatment of spheroids

**Figure 2:**
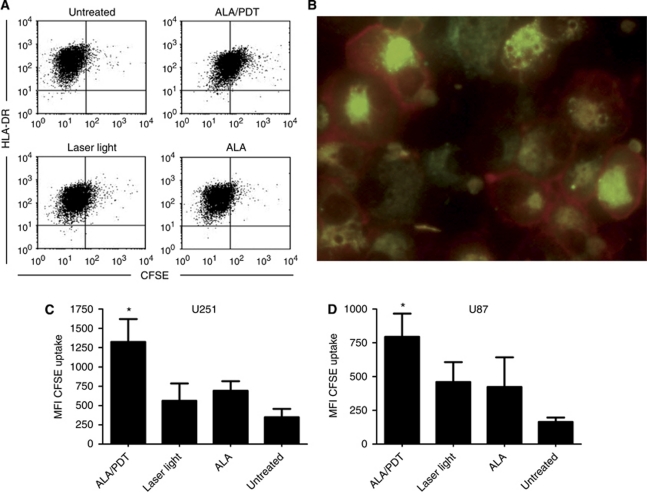
Uptake of tumour material from glioma spheroids by immature DCs. Immature DCs were co-cultured overnight with CFSE-labelled U251 (**A**–**C**) or U87 spheroids (**D**), untreated or treated with ALA/PDT, ALA-only or exposure to laser-light-only. After labelling with PE-conjugated anti-HLA-DR mAb, DCs were analysed by flow cytometry (**A**, **C** and **D**) or fluorescence microscopy (**B**). Antigen uptake by the DCs was assessed by determining the CFSE MFI (CFSE-labelled tumour material) of the HLA-DR^+^ DC population (**C** and **D**). Data are presented as mean±s.e.m for *n*=4. Statistically significant differences compared to untreated control spheroids are indicated; ^*^P⩽0.05.

To determine uptake of tumour material from glioma spheroids, CFSE-labelled ALA/PDT-treated spheroids or control spheroids were co-cultured with immature DCs for 16 h, and antigen uptake by the DCs was subsequently quantified by flow cytometry. Pre-treatment of spheroids with ALA/PDT resulted in a significant uptake of tumour material by the DCs from U251 (Figures 2A–C; 1324.0±297.6 *vs* 349.3±108.0 mean fluorescence intensity (MFI) FITC; *n*=4, *P*=0.0217) as well as from U87 spheroids (Figure 2D; 794.3±172.1 *vs* 164.3±33.1 MFI FITC; *n*=4, *P*=0.0114) compared to untreated control spheroids. Spheroids that had been treated with ALA-only or laser-light-only showed no such activity. Fluorescence microscopy of DCs revealed perinuclear localisation of the fluorescent tumour material (Figure 2B).

### ALA/PDT-treated spheroids induce maturation of DCs

**Figure 3:**
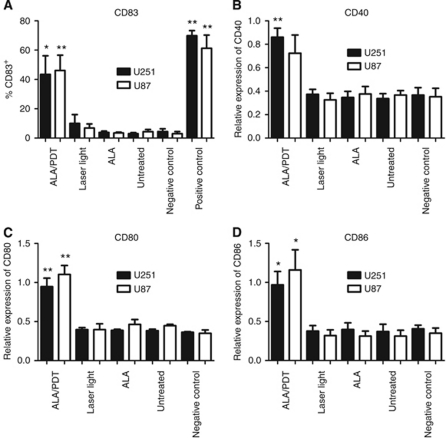
Dendritic cell maturation-inducing activity of glioma spheroids. Immature DCs were co-cultured with untreated, ALA/PDT-treated, ALA-only-treated, laser-light-only-treated or in the absence (negative control) of U251 (white bars) or U87 spheroids (black bars). Cultures supplemented with 1000 U ml^−1^ TNF*α* served as positive controls. After 3 days of culture, the frequency of CD83^+^ cells (**A**) and the relative expression of CD40, CD80 and CD86 (**B**–**D**) were determined by flow cytometry. Expression of CD40, CD80 and CD86 was normalised to the respective MFI values of the positive control (TNF*α*-induced maturation), which was set to 1. Data are presented as mean±s.e.m for *n*⩾3. Statistically significant differences compared to negative control cultures in the absence of spheroids are indicated; ^**^P⩽0.01, ^*^P⩽0.05.

To study the influence of glioma spheroids on DC maturation, immature DCs were co-cultured for 3 days in the presence or absence of ALA/PDT-treated or control spheroids. Subsequently, the expression of the maturation marker CD83 and the co-stimulatory molecules CD40, CD80 and CD86 was analysed. Cultures with TNF*α* for 3 days served as positive controls for DC maturation. In the presence of ALA/PDT-treated spheroids, a significant proportion of DCs had matured as evident by an increased frequency of CD83^+^ cells compared to control cultures in the absence of maturation stimuli (U251, 43.5±12.6 *vs* 4.5±1.9% CD83^+^; *n*=4, *P*=0.0223; U87, 46.1±10.4 *vs* 3.1±1.4% CD83^+^; *n*=5, *P*=0.0034). In contrast, co-culture of immature DCs with control spheroids (untreated, ALA-only- or laser-light-only-treated) did not induce DC maturation (Figure 3A). Similar results were obtained when the expression of the co-stimulatory molecules was analysed. Only after co-culture with ALA/PDT-treated spheroids, but not with the control spheroids, a significant upregulation of CD40, CD80 and CD86 on the DCs compared to control cultures without maturation stimuli was observed (Figure 3B).

**Figure 4:**
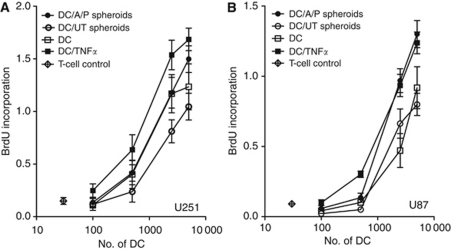
Allostimulatory activity of DCs matured in the presence of glioma spheroids. Graded dose of immature DCs (baseline control), TNF*α*-matured DCs (positive control) or immature DCs that had been co-cultured for 3 days with ALA/PDT-treated or untreated spheroids of the cell lines U251 (**A**) or U87 (**B**) were co-cultured with allogeneic T cells and after 6 days bromodeoxyuridine incorporation was determined. Data are presented as mean±s.e.m for *n*=3.

Functional activity of DCs matured in the presence or absence of ALA/PDT-treated or untreated spheroids of the cell lines U251 (Figure 4A) or U87 (Figure 4B) was evaluated in an allogeneic mixed leukocyte reaction. Control immature DCs that were neither exposed to spheroids nor to the maturation-inducing TNF*α* revealed allostimulatory activity, which was comparable to that of DCs co-cultured with untreated spheroids (*P*>0.05 (ANOVA); *n*=3), with a trend towards reduced allostimulatory activity of the DCs after co-culture with untreated U251 spheroids (*P*=0.0644). When maturation of DCs was induced with TNF*α*, allostimulatory activity increased significantly compared to the immature DCs (*P*=0.0094 for Figure 4A and *P*=0.0004 for Figure 4B (ANOVA); *n*=3). Increased allostimulatory activity of DCs was also observed after co-culture with ALA/PDT-treated U251 (*P*=0.0014 (ANOVA); *n*=3) or U87 spheroids (*P*<0.0001 (ANOVA); *n*=3) compared to co-cultures with untreated spheroids. Co-cultures of DCs with ALA/PDT-treated U87 spheroids reached allostimulatory activity levels similar to TNF*α*-matured DCs (*P*>0.05 (ANOVA); *n*=3). For U251 spheroids, ALA/PDT treatment could compensate the reduction of allostimulatory activity observed after co-culture of immature DCs with untreated spheroids and activity increased to levels comparable to the immature DCs (*P*>0.05 (ANOVA); *n*=3). Thus, for both cell lines an increased functional activity of DCs after co-culture with ALA/PDT-treated spheroids was observed, which is consistent with the induction of DC maturation.

### ALA/PDT treatment of glioma spheroids induces HSP-70, which mediates uptake of tumour antigens and maturation of DCs

Heat-shock protein-70 has been shown to be upregulated on the surface of mouse squamous cell carcinoma cells after photofrin-mediated PDT and to contribute to immune activation (Korbelik *et al*, 2005; Korbelik and Sun, 2006). Therefore, a possible involvement of HSP-70 in the effects of ALA/PDT on DCs was addressed.

**Figure 5:**
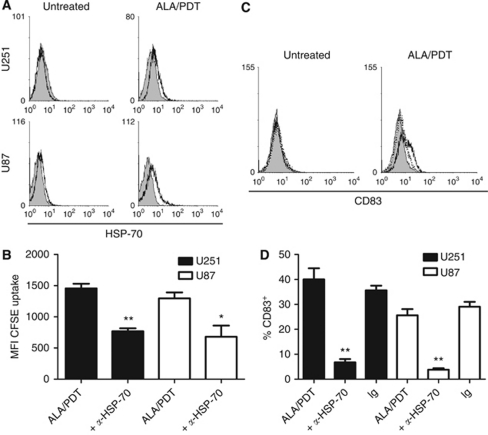
Heat-shock protein-70 surface expression contributes to antigen uptake and DC maturation induced by ALA/PDT-treated glioma spheroids. Heat-shock protein-70 surface expression (**A**) was determined on untreated and ALA/PDT-treated U251 and U87 spheroids by flow cytometry (open histograms). Negative control stainings are indicated by grey histograms. To study the influence of HSP-70 on antigen uptake (**B**) and DC maturation (**C** and **D**), untreated or ALA/PDT-treated U251 (black bars) or U87 spheroids (white bars) were pre-treated with HSP-70-specific polyclonal antibody before co-culture with the immature DCs as indicated. Pre-treatment with an irrelevant polyclonal antibody served as negative control (Ig, **D**). After 16 h (**B**) or 3 days of co-culture (**C** and **D**), antigen uptake and the frequency of CD83^+^ mature DCs were determined by flow cytometry, respectively. Uptake of fluorescently labelled tumour material by the DCs (**B**) from the spheroids is shown as mean±s.e.m of MFI values for *n*=3. Frequencies of CD83^+^ cells (**D**) are shown as mean±s.e.m for *n*⩾3. Statistically significant differences compared to assays without HSP-70 blocking are indicated; ^**^P⩽0.01, ^*^P⩽0.05. CD83 stainings of DCs after co-culture with untreated and ALA/PDT-treated U87 spheroids (open histograms) are shown in (**C**). Full lines indicate co-cultures performed with, dotted lines without HSP-70-specific blocking. Grey histograms indicate the negative control stainings.

When HSP-70 surface expression was compared for untreated and ALA/PDT-treated U251 and U87 spheroids, a clear upregulation of surface HSP-70 was evident for the treated spheroids. Heat-shock protein-70 was absent or expressed at only very low levels on the untreated spheroids (Figure 5A, representative results of three independent experiments).

Blocking of HSP-70 on the ALA/PDT-treated spheroids before assessing uptake of tumour material from the spheroids inhibited tumour antigen uptake significantly (U251, 1456.0±73.7 *vs* 769.7±46.3 MFI FITC; *n*=3, *P*=0.0014; U87, 1295.0±95.3 *vs* 679.0±180.5 MFI FITC; *n*=3, *P*=0.0393; Figure 5B). Moreover, blocking of HSP-70 also inhibited the DC maturation induced in co-cultures of immature DCs and ALA/PDT-treated U251 or U87 spheroids as indicated by a significant decrease in the frequency of CD83^+^ mature DCs (U251, 40.0±4.5 *vs* 6.7±1.3% CD83^+^; *n*=3, *P*=0.002; U87, 25.6±2.5 *vs* 3.8±0.6% CD83^+^; *n*=3, *P*=0.0006; Figures 5C, D). In contrast, no reduction in the frequency of mature CD83^+^ DCs was observed when ALA/PDT-treated U251 or U87 spheroids were treated with a control immunoglobulin before co-culture with the immature DCs (Figure 5D).

## Discussion

There is clear evidence for an immunological component in the anti-tumoural effect of PDT from extracranial tumours (Castano *et al*, 2006). Immediately after PDT, there is immigration of neutrophils into the tumour (Krosl *et al*, 1995), and depletion of neutrophils before treatment abrogates the curative effect (de Vree *et al*, 1996; Korbelik and Cecic, 1999), suggesting an important contribution of innate immunity to the efficacy of PDT. However, results from immunodeficient NOD/SCID mice, lacking B and T cells, and nude mice, lacking T cells only (Canti *et al*, 1994; Korbelik *et al*, 1996; Hendrzak-Henion *et al*, 1999; Preise *et al*, 2009), indicate an essential role of adaptive T-cell immunity as well, with CD8^+^ cytotoxic T cells as main effectors and a supportive role for CD4^+^ T-helper cells (Hendrzak-Henion *et al*, 1999; Korbelik and Cecic, 1999; Korbelik and Dougherty, 1999; Preise *et al*, 2009). Moreover, Mroz *et al* (2010) identified antigen-specific cytotoxic T cells after benzoporphyrin-derivative/PDT treatment in a CT26.CL25 colon carcinoma model. Furthermore, a recent case report suggests that an immune response contributes to tumour eradication also in humans: CD4^+^ and CD8^+^ T-cell infiltrates have been observed in lesions after Fotolon-mediated PDT of recurrent angiosarcoma, which underwent remission after therapy (Thong *et al*, 2007).

For such effector T-cell responses to occur, immature DCs have to migrate to the tumour, take-up tumour antigens and respond to local danger signals with maturation, before they will engage in T-cell activation in the draining lymph nodes in the afferent phase of cellular adaptive immunity (Ueno *et al*, 2010). Consistent with this view, Preise *et al* (2009) reported higher recurrence rates of CT26 colon carcinomas treated with WST11-mediated PDT after DC depletion. When the migration of human immature DCs to glioma spheroids was analysed here, ALA/PDT treatment resulted in significantly increased attraction of the DCs towards the tumour cells. Moreover, the immature DCs took up tumour material from the ALA/PDT-treated spheroids efficiently, whereas they did not from untreated tumour spheroids or spheroids that had only been treated with ALA or laser light alone. Thus, two of the initial steps of the afferent phase of adaptive tumour immunity are promoted by ALA/PDT.

The efficiency of PDT-treated tumour cells as vaccines for induction of anti-tumoural immunity has been shown for SCVII squamous cell carcinoma, Lewis lung carcinoma, EMT6 mammary sarcoma, P815 mastocytoma and C6 rat glioma models using photofrin, benzoporphyrin derivative or haematoporphyrin as photosensitisers (Gollnick *et al*, 2002; Korbelik and Sun, 2006; Shixiang *et al*, 2010). This implies that the PDT-treated cancer cell vaccines are recognised and taken up by the DCs. Indeed, Korbelik and Sun (2006) reported that the vaccine cells were intermixed with DC 1 h after injection, suggesting active migration of DC towards the tumour vaccines *in vivo*. Evidence for uptake of tumour antigens after PDT also comes from the intratumoural injection of DCs following PDT treatment: improved survival of animals and induction of tumour-specific immunity indicate that the DCs have taken up tumour antigens (Jalili *et al*, 2004; Saji *et al*, 2006; Sur *et al*, 2008). Furthermore, when immature DCs were co-cultured *in vitro* with CT-26 colon carcinoma cells subjected to photofrin/PDT, enhanced uptake of tumour material by the DCs was detected (Jalili *et al*, 2004).

Attraction of DCs and uptake of tumour antigens alone, however, are not sufficient for the initiation of an immune response, but may result in immunological tolerance (Green *et al*, 2009; Tisch, 2010; Ueno *et al*, 2010). An activating danger signal inducing DC maturation is required. Here, ALA/PDT-treated spheroids provided such a maturation-inducing stimulus. When immature DCs were co-cultured with ALA/PDT-treated spheroids, maturation of DCs was induced as indicated by the induction of the marker for mature DCs, CD83 and upregulation of the co-stimulatory molecules CD40, CD80 (B7.1) and CD86 (B7.2). Importantly, these changes in the expression of immunorelevant molecules were associated with an increased T-cell stimulatory activity of the DCs. From the efficacy of PDT-generated vaccines, which was superior compared to vaccines generated using UV or ionising irradiation (Gollnick *et al*, 2002; Korbelik and Sun, 2006), and from the efficacy of intratumourally injected DCs following PDT (Jalili *et al*, 2004; Saji *et al*, 2006; Sur *et al*, 2008) as well as from increased frequencies in IFN*γ*-secreting cells and the generation of tumour-specific CTL in the treated animals reported by others, it can be concluded that DCs have undergone maturation owing to PDT treatment of the tumour cells in these studies as well. This has been confirmed by *in vitro* analyses showing induction of MHC class II molecules, CD80, CD86 and IL-12 in co-culture experiments of DCs and PDT-treated mouse tumour cells (Gollnick *et al*, 2002; Jalili *et al*, 2004) or DCs and acid-eluted material from PDT-treated C6 rat glioma cells (Shixiang *et al*, 2010).

The factors and mechanisms involved in promoting attraction, antigen uptake and maturation of DCs following PDT are not well understood. Depending on the photosensitiser, its location within the cell and dose, the cell type and oxygen concentration, PDT results in apoptotic or necrotic cell death (Castano *et al*, 2006). The protocol used in this study mainly induced apoptosis as determined by TUNEL staining (Etminan *et al*, 2011). Induction of apoptosis after ALA/PDT treatment of GB cells has also been reported by Inoue *et al* (2007). Apoptotic cells release several chemoattractive factors, including nucleotides, lipids and chemokines, which may attract DCs. Changes in surface molecules on apoptotic cells, for example, expression of calreticulin or HSP-70 and -90, allow recognition and engulfment of apoptotic bodies (Green *et al*, 2009; Zitvogel *et al*, 2010). Although apoptotic cells have been suggested to fail to induce DC maturation and to be associated with induction of immunological tolerance, this also appears to depend on the pre-apoptotic conditioning, for example, stress, of the cells together with the sequence of the events rather than solely on apoptotic *vs* necrotic cell death. The release of inflammatory mediators, damage-associated molecular patterns and alarmins like the pro-inflammatory cytokines HMGB1 and IL-1*α* during apoptosis may contribute to DC maturation (Green *et al*, 2009; Zitvogel *et al*, 2010). It is well established that PDT results in the upregulation of inflammatory mediators, including IL-1*β*, IL-6, TNF*α*, prostaglandins and various HSPs, and glucose-regulated proteins (Gollnick *et al*, 1997; Dolmans *et al*, 2003; Jalili *et al*, 2004; Korbelik *et al*, 2005; Castano *et al*, 2006). For the glioma spheroids, we observed upregulation of HSP-70 surface expression, and when HSP-70 was blocked by antibodies, uptake of tumour antigens as well as DC maturation induced by the ALA/PDT-treated spheroids was almost completely inhibited. Korbelik *et al* (2005) and Korbelik and Sun (2006) also suggested a pivotal role of surface HSP-70 in PDT-induced immune activation: inhibitors to HSP-70, Toll-like receptors 2 and 4 or specific inhibition of NF-*κ*B blocked macrophage activation and TNF*α* release induced by photofrin-mediated PDT of SCCVII mouse squamous cell carcinoma cells. Thus, surface expression of HSP-70 appears to be a major DC maturation-inducing stimulus after PDT in mice as well as in humans. Whether other factors, including pro-inflammatory cytokines and HSP or glucose-regulated proteins, which have been reported to be upregulated on tumour cells in response to PDT (Gollnick *et al*, 1997; Dolmans *et al*, 2003; Jalili *et al*, 2004; Korbelik *et al*, 2005; Castano *et al*, 2006), are also upregulated and contribute to attraction, antigen uptake and maturation of DCs by ALA/PDT-treated GB cells is currently unknown.

For human GB, the immunological consequences of ALA/PDT have not been analysed so far. In this study, we could show that ALA/PDT treatment of GB spheroids attracts DCs and promotes uptake of tumour antigens as well as DC maturation *in vitro*. However, our study holds limitations: although the spheroid model may mimic microtumours more closely than monolayer cultures (Sutherland, 1988; Hirschhaeuser *et al*, 2010), differences between the established cell lines used here and primary tumour cells *in vitro* or the tumour, including its vasculature and stroma *in vivo*, may result in a different effect of ALA/PDT. In addition, it remains to be elucidated whether the above-described effects on DCs result in cross-presentation of tumour antigens and induction of cytotoxic T-cell-mediated anti-tumoural immunity *in vivo.* Therefore, further studies evaluating the effect of ALA/PDT on primary tumour cells as well as *in vivo* studies are needed to document efficacy of PDT in GB and the contribution of an anti-tumoural immune response to it.

In summary, like in extracerebral tissues, ALA/PDT may not only kill GB cells directly due to its phototoxic effect, but may also result in the induction of anti-tumoural immunity, mediated by the induction of HSP-70 surface expression. This immunological effect may contribute to long-sustaining responses and the potential clinical benefit of ALA/PDT for GB (Eljamel *et al*, 2008; Stummer *et al*, 2008).
